# Bcl2 inhibition of mitochondrial DNA repair

**DOI:** 10.1186/s12885-015-1594-1

**Published:** 2015-08-13

**Authors:** Maohua Xie, Paul W. Doetsch, Xingming Deng

**Affiliations:** 1 Division of Cancer Biology, Departments of Radiation Oncology, Emory University School of Medicine and Winship Cancer Institute of Emory University, Atlanta, GA 30322 USA; 2 Biochemistry, Emory University School of Medicine and Winship Cancer Institute of Emory University, Atlanta, GA 30322 USA

**Keywords:** Bcl2, APE1, Mitochondrial DNA repair, Mitochondrial DNA mutation, Carcinogenesis

## Abstract

**Background:**

Accumulation of mitochondrial DNA (mtDNA) damage could enhance the frequency of mitochondrial mutations and promote a variety of mitochondria-related diseases, including cancer. However, the mechanism(s) involved are not fully understood.

**Methods:**

Quantitative extended length PCR was used to compare mtDNA and nDNA damage in human lung H1299 cells expressing WT Bcl2 or vector-only control. mtAPE1 endonuclease activity was analyzed by AP oligonucleotide assay. mtDNA mutation was measured by single molecule PCR. Subcellular localization of Bcl2 and APE1 was analyzed by subcellular fractionation**.**

**Results:**

Bcl2, an anti-apoptotic molecule and oncoprotein, effectively inhibits the endonuclease activity of mitochondrial APE1 (mtAPE1), leading to significant retardation of mtDNA repair and enhanced frequency of mtDNA mutations following exposure of cells to hydrogen peroxide (H_2_O_2_) or nitrosamine 4-(methylnitrosamino)-1-(3-pyridyl)-1-butanone (NNK, a carcinogen in cigarette smoke). Inversely, depletion of endogenous Bcl2 by RNA interference increases mtAPE1 endonuclease activity leading to accelerated mtDNA repair and decreased mtDNA mutation. Higher levels of mtAPE1 were observed in human lung cancer cells than in normal human bronchial epithelial cells (i.e. BEAS-2B). Bcl2 partially co-localizes with APE1 in the mitochondria of human lung cancer cells. Bcl2 directly interacts with mtAPE1 via its BH domains. Removal of any of the BH domains from Bcl2 abolishes Bcl2’s capacity to interact with mtAPE1 as well as its inhibitory effects on mtAPE1 activity and mtDNA repair.

**Conclusions:**

Based our findings, we propose that Bcl2 suppression of mtDNA repair occurs through direct interaction with mtAPE1 and inhibition of its endonuclease activity in mitochondria, which may contribute to enhanced mtDNA mutations and carcinogenesis.

**Electronic supplementary material:**

The online version of this article (doi:10.1186/s12885-015-1594-1) contains supplementary material, which is available to authorized users.

## Background

Mitochondria contain their own genome (i.e. mitochondrial DNA, mtDNA), which comprises a small, self-replicating DNA molecule present in multiple copies in the mitochondrial matrix [1]. The human mitochondrial genome is a tiny 16.6 kb circle containing only 37 genes. Thirteen of these genes encode proteins, and the remaining 24 consist of 2 ribosomal RNAs (rRNAs) and 22 tRNAs that are used for translation of those 13 polypeptides [2]. In contrast to nuclear DNA (nDNA), mtDNA is uninterruptedly replicated even in terminally differentiated cells. mtDNA is much more susceptible to oxidative damage than the nuclear genome, presumably because it lacks protective histones and due to its proximity to reactive oxygen species (ROS) endogenously generated by the mitochondrial electron transport complexes [3, 4]. Such damage includes several dozen oxidized bases, apurinic/apyrimidinic (AP) sites, and oxidation products of AP sites leading to DNA strand breaks [5]. Nitrosamine 4-(methylnitrosamino)-1-(3-pyridyl)-1-butanone (NNK) is the most potent carcinogenic constituent in cigarette smoke that can induce DNA damage, including AP sites in nDNA [6–8]. The mtDNA is very sensitive to oxidative stress-induced damage [9]. It is currently unclear whether NNK induces mtDNA damage.

The rate of mtDNA mutations can be more than two orders of magnitude higher than that of nDNA [10]. Somatic mutations of mtDNA are potentially more harmful for cell functions compared to somatic damages of nDNA. Consequently, the DNA repair systems in the mitochondria may be more important than in the nuclei, especially in non-dividing cells [11]. Accumulated mtDNA mutations have been proposed to be associated with cancer [12], neurodegenerative disorders [13], diabetes [14], and premature aging [15–18]. Human apurinic/apyrimidinic endonuclease 1 (APE1) is a major component of the base excision repair (BER) pathway of AP sites [19]. Two functionally independent domains of the protein were characterized and determined to perform two different activities: the N terminus domain is principally defined to possess redox activity, whereas the C terminus region exerts its enzymatic activity on the repair of AP sites [19, 20]. APE1 specifically binds to AP sites and initiates repair by incision of the 5’phosphodiester bond to generate a 3’ hydroxyl terminus, which serves as the primer required for gap-filling in the BER repair pathway [19]. In addition to nuclear localization, APE1 has been reported to be localized in mitochondria in various types of cells, including lung cancer cells [19, 21–23]. Because repair of oxidative mtDNA damage also occurs through the BER pathway in various cell types [24, 25], APE1 has been considered to play a central role in repairing AP sites in both nDNA and mtDNA [26].

Bcl2 is a major anti-apoptotic molecule in the Bcl2 family that can suppress apoptosis to prolong cell survival [27]. In addition to its survival activity, Bcl2 can also inhibit the repair of various types of DNA damage, including AP sites [7] and DNA double strand breaks (DSBs) [28], by negatively regulating APE1-mediated BER and Ku-mediated nonhomologous end joining (NHEJ) pathways. We previously demonstrated that Bcl2 potently suppresses the repair of NNK-induced AP sites in the nucleus through direct interaction with nuclear APE1 and subsequent inhibition of its endonuclease activity [7, 29]. Since the majority of Bcl2 is localized in mitochondria [30–32], Bcl2 may also play an important role in the regulation of mtDNA repair. In the present report, we show that Bcl2 suppresses mtDNA repair through direct interaction with APE1 in mitochondria via its BH domains and inhibition of mtAPE1 endonuclease activity, leading to increased frequency of mtDNA mutations following exposure of cells to H_2_O_2_ or NNK. These findings identify a novel role for Bcl2 in regulating mtDNA repair and mtDNA mutagenesis.

## Methods

### Materials

Bcl2, APE1, PCNA, prohibitin and tubulin antibodies were purchased from Santa Cruz Biotechnology (Santa Cruz, CA). MTT cell growth kit was obtained from Sigma (St. Louis, MO). QIAamp DNA isolation kit was purchased from Qiagen (Chatsworth, CA). 4,6-diamidino-2-phenylindole (DAPI), QD605 goat anti-rabbit IgG conjugate (red), QD705 goat anti-mouse IgG conjugate (green) and Pico Green dsDNA Quantitation kit were obtained from Invitrogen (Carlsbad, CA). HEX-5ʹ-end-labeled 26-mer duplex oligonucleotide (5ʹ-AAT TCA CCG GTA CCF CCT AGA ATT CG-3’) was purchased from IDT Technologies (Coralville, IA). LA PCR Kit and TaKaRa ExTak PCR kit were obtained from Clontech Laboratories, Inc (Mountain View, CA). All of the reagents used were obtained from commercial sources unless otherwise stated.

### Cell lines, plasmids, and transfections

H1299 and H460 cells were maintained in RPMI 1640 with 5 % bovine serum and 5 % fetal bovine serum. These cell lines were employed for the described experiments without further authentication. WT and Bcl2 BH deletion mutants were created and stably expressed in H1299 cells as previously described [28]. The expression levels of exogenous Bcl2 were analyzed by Western blot analysis. Three separate clones expressing similar amounts of exogenous Bcl2 were selected for further analysis.

### Preparation of cell lysates

Cells were washed with 1xPBS and resuspended in ice-cold 1 % CHAPS lysis buffer (1 % CHAPS, 50 mM Tris [pH 7.6], 120 mM NaCl, 1 mM EDTA, 1 mM Na_3_VO_4_, 50 mM NaF, and 1 mM β-mercaptoethanol) with a cocktail of protease inhibitors (EMD Biosciences). Cells were lysed by sonication and centrifuged at 14,000 × g for 10 min at 4 °C. The resulting supernatant was collected as the total cell lysate.

### Subcellular fractionation

Cells were washed twice in PBS and then resuspended in isotonic mitochondrial buffer (210 mM mannitol, 70 mM sucrose, 1 mM EGTA, 10 mM Hepes, pH 7.5) containing protease inhibitor mixture set I (Calbiochem). The resuspended cells were homogenized with a polytron homogenizer operating for four bursts of 10 s each at a setting of 5. The mitochondrial, light membrane, cytosol and nuclear fractions were isolated as we previously described [33]. Protein from each fraction was analyzed by Western blot.

### Quantum dot-based immunofluorescence (QD-IF)

QD-IF was performed as described previously [34]. Briefly, cells were washed with 1 × PBS, fixed with cold methanol and acetone (1:1) for 10 min, and then blocked with 1 % normal goat serum for 60 min at room temperature. The cells were incubated simultaneously with mouse Bcl2 and rabbit APE1 primary antibody overnight at 4 °C. After washing, the samples were incubated with QD secondary antibody conjugates (QD 605 goat F(ab’)2 anti-rabbit IgG, red; QD 705 goat F(ab’)2 anti-mouse IgG, green 1:50 dilution) in a cocktail solution at room temperature for 60 min. Cell nuclei were counterstained with DAPI. Mouse and rabbit IgG were used as negative controls. QD imaging and quantification procedures were performed as described previously [34]. The Nuance™ fluorescence microscope system (CRi consolidated with Caliper, a PerkinElmer company, Hopkinton, MA) was used for quantification of the QD signals. All cubed image files were collected from culture cells at 10 nm wavelength intervals from 420–720 nm, with an auto exposure time per wavelength interval at 200 ~ 400× magnification. Taking the cube with a long wavelength band pass filter allowed transmission of all emission wavelengths above 420 nm. Both separated and combined QD images were obtained after establishing the QD spectral library and unmixing the image cube. For each cell sample, 10 cubes were taken. The background signal was removed for accurate quantification of the QD signals. Cells were observed and signal was quantified by an Olympus microscope IX71 with a CRi Nuance spectral imaging and quantifying system (CRi Inc., Woburn, MA) [34, 35]. The co-localization of Bcl2 and APE1 was quantified by Nuance imaging software (Caliper/PerkinElmer), 10 randomly selected fields on the cell slides were calculated.

### AP oligonucleotide assay for mtAPE1 endonuclease activity

Intact mitochondria were isolated as we described previously [36]. The isolated mitochondria from cells were resuspended in 0.5 % NP-40 lysis buffer and rocked for 60 min prior to centrifugation at 17,530 × *g* for 10 min at 4 °C. The resulting supernatant was used as mitochondrial extract for the mtAPE1 activity assay. APE1 activity was analyzed by measuring incision of a HEX-5ʹ-end-labeled 26-mer duplex oligonucleotide substrate containing a synthetic tetrahydrofuran (THF, F) AP site as described previously [37]. Reaction mixtures (20 μl) containing 1 μg mitochondrial extract, 5 pmol of HEX-5ʹ end labeled, double-stranded THF oligonucleotide, 50 mM HEPES, 50 mM KCl, 10 mM MgCl2, 1 μg/ml BSA, and 0.05 % Triton X-100 (pH 7.5) were incubated at 37 °C for 15 min. The reaction was stopped by the addition of 20 μl formamide and 10 mM EDTA. Samples were separated by a 20 % polyacrylamide gel containing 7 M urea. The bands of 14-mer (cleavage product) and 26-mer (uncleaved substrate) oligonucleotides were visualized by Typhoon 9410 imager system and quantified using ImageQuant^™^ software (Molecular Dynamics). The AP endonuclease activity was calculated by the formula: 14 ‐ mer/(14 ‐ mer + 26 ‐ mer) × 100.

### Analysis of mtDNA and nDNA damage by quantitative extended length PCR (QPCR)

mtDNA and nDNA damage was analyzed by QPCR as described previously [9, 38]. Briefly, cells in serum-free-medium were treated with H_2_O_2_ and NNK for 1 h. Cells were then washed three times and incubated in normal medium for various times. DNA was isolated with the QIAamp DNA isolation kit for QXLPCR using LA PCR Kit. Primers for mtDNA (16.2 kb): 5’ TGA GGC CAA ATA TCA TTC TGA GGG GC 3’ (sense); 5’ TTT CAT CAT GCG GAG ATG TTG GAT GG 3’ (antisense). Primers for the β-globin gene of nDNA (17.7 kb): 5’ TTG AGA CGC ATG AGA CGT GCA G 3’ (sense); 5’ GCA CTG GCT TAG GAG TTG GAC T 3’ (antisense). The PCR product was quantified by Pico Green dsDNA Quantitation kit as described [38].

### MTT cell proliferation assay

Cells were treated with H_2_O_2_ (100 μM) or NNK (200 μM) in serum-free medium for 60 min, and then washed three times with 1 × PBS. Cells were then allowed to recover in regular medium for 24 h. Cells were incubated with MTT (3-(4,5-dimethylthiazol-2-yl)-2,5-diphenyl tetrazolium bromide) at a final concentration of 2.0 μg/ml during the last hour of the recovery period, followed by lysis in 20 % SDS and 50 % DMSO in 1 × PBS buffer. Samples were measured at an absorbance of 570 nm. MTT reduction for treated samples was then normalized to that of non-treated control samples.

### Cell cycle analysis

After treatment with H_2_O_2_ or NNK, cells were washed once with ice-cold PBS and resuspended in 100 μL of ice-cold PBS. Then 900 μL of cold methanol was added to the cells, mixed gently and then incubated on ice or in a −20 °C freezer for at least 30 min. Cells were washed once with PBS and resuspended in 500 μL PBS. RNAse (100 μg/mL) was added and incubated at room temperature for 60 min. Next, 500 μL of 0.1 mg/mL propidium iodide (PI) was added to cells and incubated at room temperature for 30 min. Cell cycle was analyzed by flow cytometry as described previously [39].

### Analysis of mtDNA mutation by single molecule PCR

Single molecule PCR was applied to analyze mtDNA mutation as described [40, 41]. Cells were treated with 100 μM H_2_O_2_ or 200 μM NNK for 60 min. Cells were washed 3 times and cultured in fresh medium for 15 days. Total DNA was isolated with the QIAamp DNA isolation kit and diluted to 1:10^6^. PCR was carried out using TaKaRa ExTak PCR system according to the manufacturer’s instructions. First round PCR was carried out for 40 cycles (95 °C for 20 s, 68 °C for 2 min). 3 μl of PCR mixture was then used for second round PCR (additional 25 cycles). Primers for first round PCR: 5’ ATT CTA ACC TGA ATC GGA GG 3’ and 5’ GAT GCT TGC ATG TGT AAT CT 3’; Primers for second round PCR: 5’ AGG ACA ACC AGT AAG CTA CCC T 3’ and 5’ ACT AAG AGC TAA TAG AAA G 3’. The final PCR products were subjected to electrophoresis on 0.8 % agarose gel and purified for DNA sequencing. Mutation load was calculated as described [40, 41].

### Bcl2 silencing

Bcl2 shRNA and its control shRNA were purchased from Santa Cruz Biotechnology (Santa Cruz, CA). Hairpin sequence of Bcl2 RNA: GAT CCG TGT GGA TGA CTG AGT ACC TGA TTC AAG AGA TCA GGG ACT CAG TCA TCC ACA TTT TTG. Hairpin sequence of control shRNA: GAT CCG GAA CGG CATC AAG GTG AAC TTC AAG AGA GTT CAC CTT GAT GCC GTT CTT TTT G. For pseudovirus production, Bcl2 shRNA or control shRNA was cotransfected into 293FT cells with lentivector packaging plasmid mixture (System Biosciences, CA) using NanoJuice transfection kit (EMD Chemical, Inc.) as described [42]. After 48 h, the virus-containing media were harvested by centrifugation at 20,000 × g. H460 cells were infected with the virus-containing media in the presence of polybrene (8 μg/ml) for 24 h following which stable positive clones were selected using 1 μg /ml puromycin. The levels of Bcl2 expression were analyzed by Western blot. Specific silencing of the targeted Bcl2 gene was confirmed by at least three independent experiments.

### Statistical analysis

Significant differences between two groups were analyzed using two-sided unpaired Student’s *t*-test. A p value < 0.05 was considered statistically significant.

## Results

### mtDNA is more sensitive than nDNA to NNK or H_2_O_2_-induced damage

**Fig. 1:**
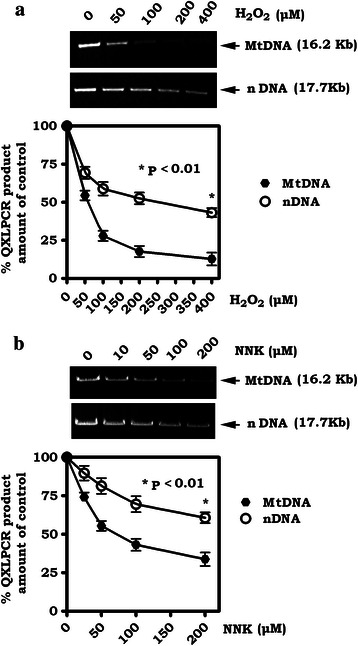
Mitochondrial DNA (mtDNA) is more vulnerable than nuclear DNA (nDNA) to damage induced by H_2_O_2_ or NNK. **a** and **b**. H1299 cells were treated with increasing concentrations of H_2_O_2_ (**a**) or NNK (**b**) for 60 min. The mtDNA or nDNA damage was measured by QXLPCR and quantified by Pico Green dsDNA Quantitation kit as described in “Methods”. Quantification data are mean ± SD from three independent experiments

To test whether NNK induces mtDNA damage, we employed quantitative amplification (QPCR) of long DNA fragments as previously described [9]. H_2_O_2_ was used as a positive control since its ability to induce mtDNA damage is well established [9]. mtDNA (a 16.2-kb mtDNA fragment) and nDNA (a 17.7-kb fragment from the β-globin loci) were analyzed by QPCR following exposure of H1299 cells to increasing concentrations of H_2_O_2_ or NNK. Results revealed that, in addition to H_2_O_2,_ NNK also induced mtDNA damage in a dose-dependent manner (Fig. 1a, b). Importantly, mtDNA was more sensitive than nDNA to NNK or H_2_O_2_-induced damage (Fig. 1a, b). Similar experiments were also performed in another human lung cancer cell line (i.e. H460) and yielded similar results (Additional file 1: Figure S1), suggesting that the effect of NNK or H_2_O_2_ on mtDNA and nDNA is a general reaction and not a cell type-specific phenomenon. This supports and extends the findings of a previous report [9]. To rule out the possibility of mtDNA pseudogenes amplified by the mitochondrial primers used, QPCR experiments using the same primers were performed in DU145 cells and the mtDNA deficient DU145ρ^0^ cells [43]. Results confirmed that mtDNA was observed only in DU145 cells but not in DU145 DU145ρ^0^ cells (Additional file 1: Figure S2).

### Expression of Bcl2 inhibits mtDNA repair induced by H_2_O_2_ or NNK leading to increased frequency of mtDNA mutations

**Fig. 2:**
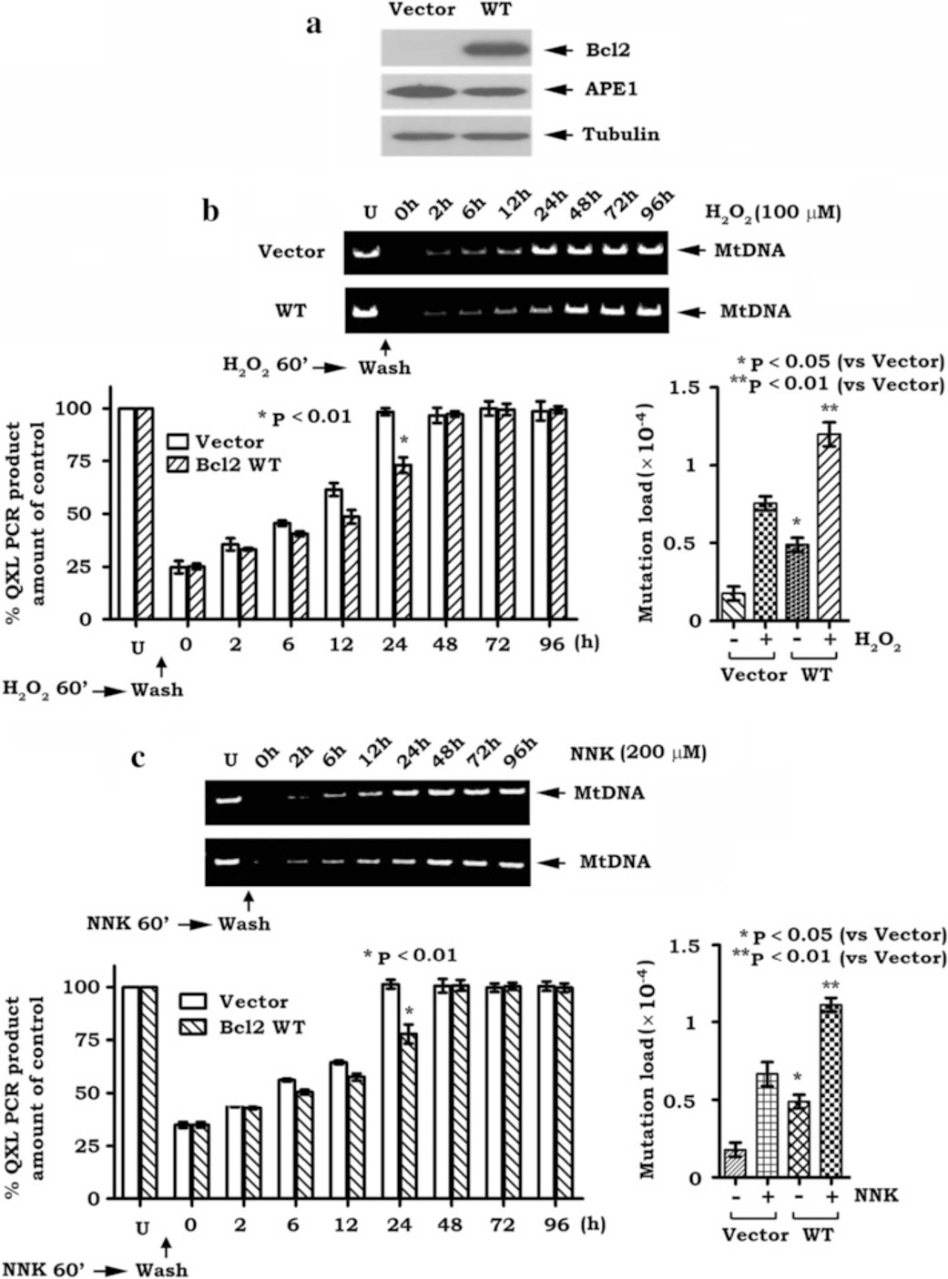
Bcl2 inhibits mtDNA repair in association with increased frequency of mtDNA mutations. **a**. Levels of Bcl2 and APE1 were analyzed by Western blot in H1299 cells expressing WT Bcl2 and vector-only control. **b** and **c**. H1299 cells expressing WT Bcl2 and vector-only control were treated with 100 μM H_2_O_2_ (b) and 200 μM NNK (c) for 60 min. Cells were then washed three times and incubated in fresh culture medium for various times as indicated. mtDNA damage or mtDNA mutation was analyzed as described in “Methods”. Quantification data are mean ± SD from three independent experiments

Bcl-2 has been implicated in the negative regulation of repair of various types of DNA damage in the nucleus [7, 28, 29, 44]. It remains unknown whether Bcl2 affects mtDNA repair since Bcl2 is mainly localized in mitochondria [45]. To determine whether Bcl2 regulates mtDNA repair, H1299 cells expressing Bcl2 or vector-only control (Fig. 2a) were treated with H_2_O_2_ (100 μM) or NNK (200 μM) for 60 min. More than 90 % of the cells remained alive for a short time (60 min) following treatment at the doses used (data not shown). Cells were then washed and incubated in normal cell culture medium for various times as indicated. mtDNA damage was analyzed by QPCR. Intriguingly, there was no significant difference in mtDNA damage between cells expressing Bcl2 and vector control cells initially following H_2_O_2_ or NNK exposure (Fig. 2b). As compared to vector-only control cells, the repair of H_2_O_2_ or NNK-induced mtDNA damage was significantly delayed in cells expressing WT Bcl2 (i.e. 48 h vs. 24 h) (Fig. 2b, c), indicating that Bcl2 inhibits mtDNA repair. Additionally, the effect of H_2_O_2_ or NNK on cell proliferation or cell cycle was analyzed. Results indicate that H_2_O_2_ or NNK at 100 μM reduced proliferation and enhanced the proportion of H1299 and H460 cells in S and G2 phases (Additional file 1: Figure S3). It has been previously reported that the APE1 expression level varies within the cell cycle in NIH 3 T3 cells and that APE1 regulates the proliferation and migration of pancreatic cancer cells [46, 47]. It is possible that, in addition to mtDNA repair, APE1 may play a role in regulating cell proliferation or cell cycle after exposure of cells to H_2_O_2_ or NNK. To explore whether Bcl2 inhibition of mtDNA repair enhances the frequency of mtDNA mutations, single molecule PCR was employed for the analysis of mtDNA mutations as described in Materials and Methods [41]. The advantage of single molecule PCR is that PCR driven-errors are excluded [40, 41], thus, the sequence results represent the true mtDNA mutation load. As shown in Fig. 2b and c, right panels, expression of Bcl2 not only enhanced mtDNA mutations but also significantly increased H_2_O_2_ or NNK-induced mtDNA mutation load.

### Bcl2 co-localizes and interacts with mtAPE1 via BH domains on mitochondrial membranes

**Fig. 3:**
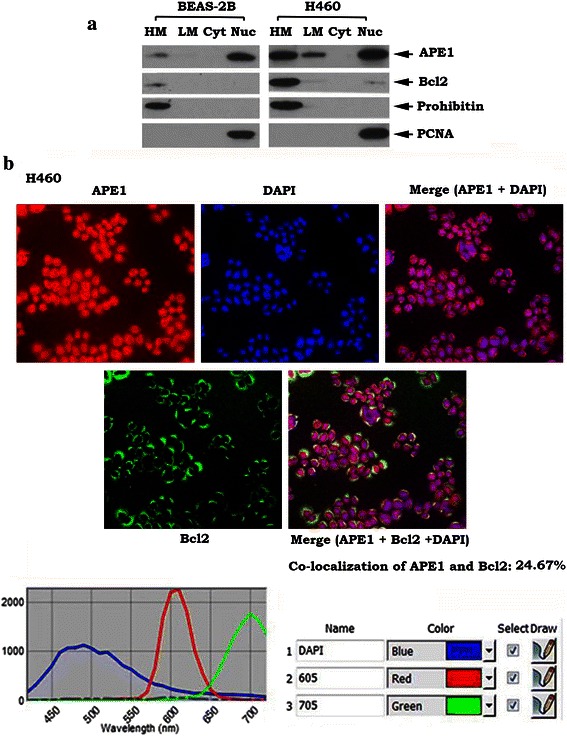
Bcl2 is co-localized with APE1 via BH domains in mitochondria. **a**. Subcellular fractionation was performed in H460 and BEAS-2B cells to isolate heavy membrane (HM), light membrane (LM), cytosol (Cyt) and nuclear (Nuc) fractions. Bcl2 and APE1 in each fraction were analyzed by Western blot. Prohibitin and PCNA were used as mitochondrial and nuclear markers, respectively. **b**. APE1 and Bcl2 were analyzed in H460 cells expressing high levels of endogenous Bcl2 and APE1 using QD-IHF and quantified as described in “Methods”. Co-localization of APE1 and Bcl2 in mitochondria was analyzed and quantified with Nuance software in 10 randomly selected fields

APE1 functions as an AP site repair enzyme and is mainly localized in the nucleus [19, 48]. A previous study indicated that APE1 has “extranuclear” localizations, including mitochondria and endoplasmic reticulum (ER), in various cells, including lung cancer, hepatocellular carcinoma and colorectal carcinoma cells [19]. Intriguingly, the extranuclear localization of APE1 is associated with poor prognosis of patients with cancer [19, 49, 50]. Our data show that, in addition to the nucleus, significantly higher levels of APE1 were observed in heavy membranes (HM) that contain mitochondrial membranes, and light membranes (LM) that contain ER in human lung cancer H460 cells compared to normal human bronchial epithelium (BEAS-2B) cells (Fig. 3a). Intriguingly, in addition to nuclear localization, APE1 also partially co-localized with MitoTracker (Additional file 1: Figure S4), providing additional evidence of mitochondrial localization of APE1 in human lung cancer cells. Because Bcl2 was found to mainly localize in the HM fraction (Fig. 3a), this indicates that APE1 may co-localize with Bcl2 in mitochondria. Prohibitin, an exclusively mitochondrial protein [51], was detected only in the HM fraction that contains mitochondrial membranes, while proliferating cell nuclear antigen (PCNA), a nuclear marker [52], was detected exclusively in the nuclear fraction (Nuc) (Fig. 3a), indicating that the fractionation procedure did not cause cross-contamination between these organelles.

To further confirm the co-localization of APE1 and Bcl2, a quantum dot-based immunofluorescence (QD-IF) technology was employed. Quantum dots (QDs) are nanoscale particles made from inorganic semiconductors that can produce different fluorescence signals depending on their size and components [34]. The advantage of this approach is that it allows for quantification of several biomarkers simultaneously on the same tissue slide [53]. QD-IF studies revealed that APE1 is localized in both the nucleus and cytoplasm in H460 cells because the extranuclear portion of APE1 could be clearly observed in the merged image of APE1 and DAPI (Fig. 3b, upper right panel). Analysis of QD images by Nuance imaging software revealed that 24.67 % of APE1 was co-localized with Bcl2 in the cytoplasm (mainly on mitochondria; Fig. 3b, lower right panel). These findings suggest a potential role of Bcl2 in regulating mtAPE1 function in mitochondria.

**Fig. 4:**
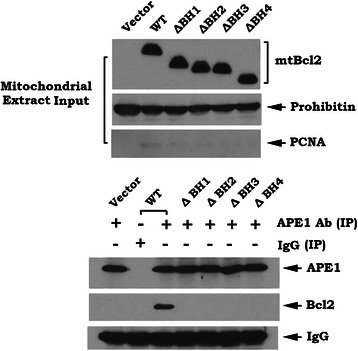
Bcl2 interacts with mtAPE1 via its BH domains in mitochondrial extracts. Co-immunoprecipitation (co-IP) experiments were performed in mitochondrial extracts isolated from H1299 cells expressing WT or each of the Bcl2 deletion mutants using APE1 antibody. Normal rabbit IgG was used for negative control for co-IP. APE1-associated Bcl2 and total mtAPE1 in mitochondrial extracts were analyzed by Western blot

To investigate whether Bcl2 interacts with APE1 via its BH domains on mitochondria, co-immunoprecipitation (co-IP) experiments were performed in isolated mitochondrial extracts from H1299 cells expressing WT or each of the BH deletion mutants using agarose-conjugated APE1 antibody. Intriguingly, APE1 interacted with WT Bcl2 protein but not with any of the BH-deleted Bcl2 mutants in isolated mitochondrial extract (Fig. 4). These findings demonstrate that mitochondrial APE1 is able to associate with Bcl2 in a BH-domain dependent manner.

### BH domains are required for the inhibitory effects of Bcl2 on mtAPE1 activity and mtDNA repair

**Fig. 5:**
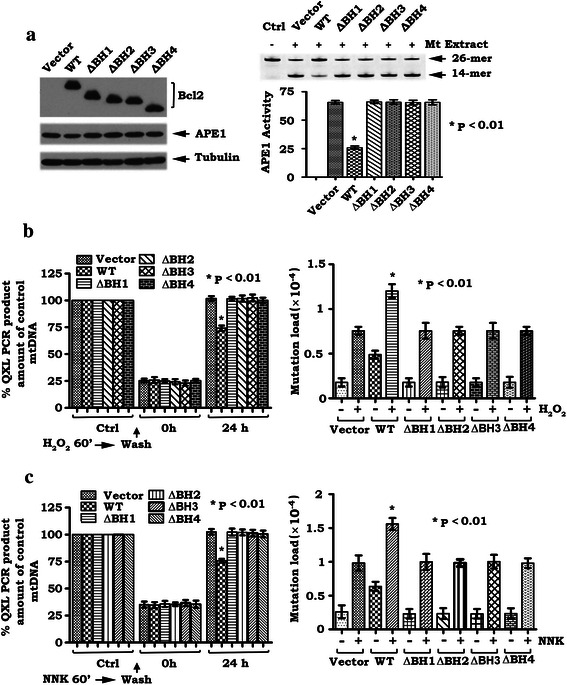
BH domains are required for Bcl2 suppression of mtAPE1 activity, mtDNA repair and enhancement of mtDNA mutations. **a**. Expression levels of Bcl2 and APE1 in H1299 cells expressing WT or each Bcl2 deletion mutant were analyzed by Western blot. HEX-labeled 26-mer AP site mimetic oligonucleotides (substrate) were incubated with mitochondrial extract isolated from H1299 cells expressing WT or each Bcl2 deletion mutant. APE1 endonuclease activity (cleavage of substrate) was analyzed by Typhoon 9410 imager system as described in “Methods”. **b** and **c**. H1299 cells expressing WT or each of the Bcl2 deletion mutants were treated with 100 μM H_2_O_2_ (**b**) and 200 μM NNK (**c**) for 60 min. Cells were then washed three times and incubated in fresh culture medium for the indicated times. mtDNA damage or mtDNA mutation was analyzed as described in “Methods”. Quantification data are mean ± SD from three independent experiments

To test whether Bcl2 affects mtAPE1 activity, mitochondrial extract was generated from H1299 cells expressing WT or each of the BH deletion mutants and then incubated with HEX-5ʹ-end-labeled 26-mer duplex oligonucleotide substrate. APE1 activities in mitochondria from H1299 cells expressing WT or each BH deletion mutant were analyzed as described in “Materials and Methods”. The cleaved 14-mer product fragment reflects AP endonuclease activity while the uncleaved 26-mer oligonucleotide correlates to lack of endonuclease activity. As shown in Fig. 5a, a decreased level of AP endonuclease activity (i.e. smaller amount of cleaved 14-mer product and greater amount of uncleaved 26-mer oligonucleotide) was observed in the mitochondrial extract from H1299 cells expressing WT Bcl2 when compared to cells expressing each BH deletion mutant or vector-only control. These findings suggest that mitochondrial Bcl2 can suppress mtAPE1 activity. The inhibitory effect of Bcl2 on mtAPE activity requires its BH domains (i.e. APE1 binding site). Importantly, BH domains are also required for Bcl2 suppression of mtDNA repair and enhancement of mtDNA mutation frequency (Fig. 5b, c).

### Depletion of endogenous Bcl2 by RNA interference results in increased mtAPE1 activity and accelerated mtDNA repair leading to reduced frequency of mtDNA mutations

**Fig. 6:**
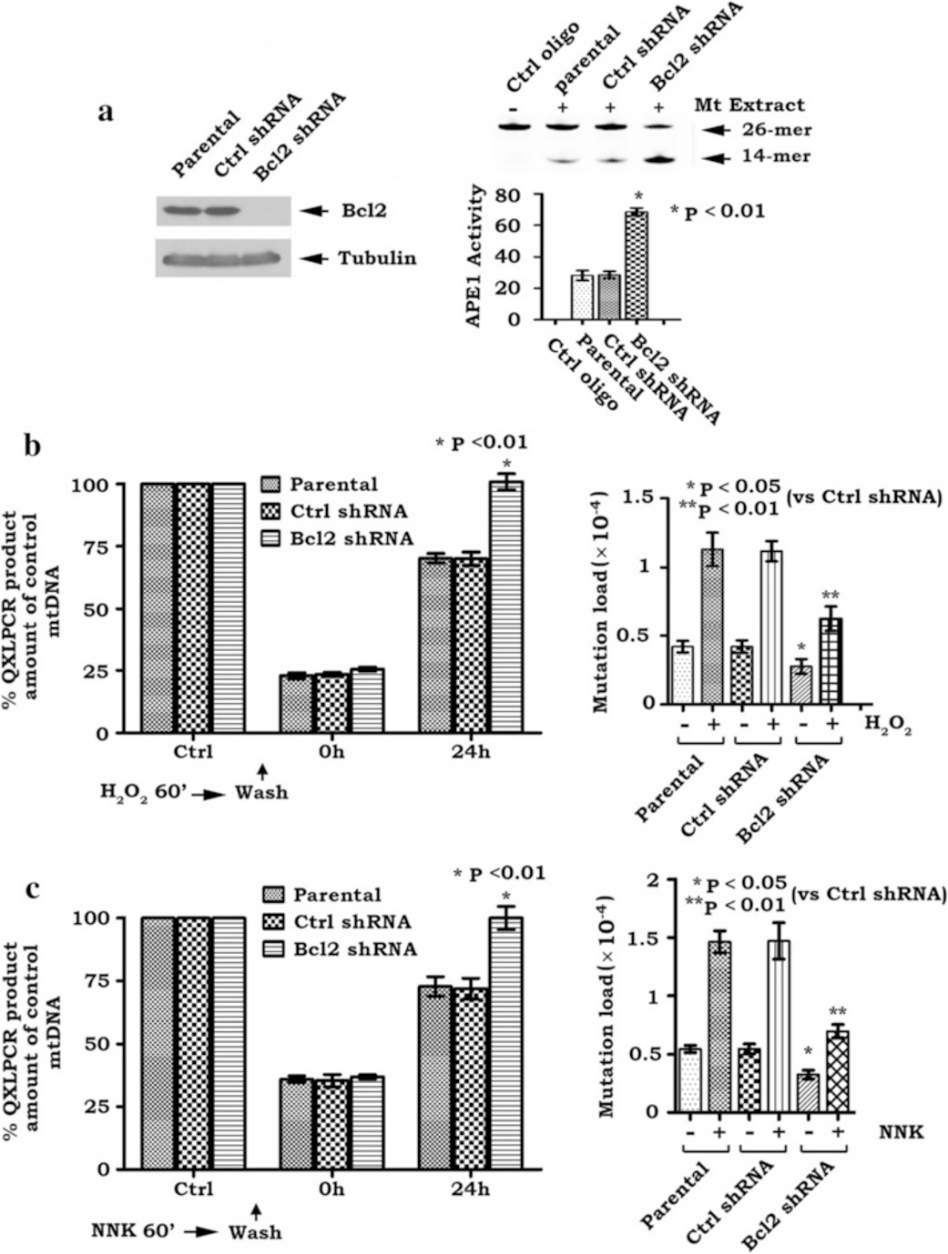
Depletion of Bcl2 by RNAi enhances mtAPE1 activity and promotes mtDNA repair in association with deceased frequency of mtDNA mutations. **a**. Bcl2 shRNA or control shRNA was transfected into H460 cells. Bcl2 expression was analyzed by Western blot. APE1 endonuclease activity was compared in H460 cells expressing Bcl2 shRNA or control shRNA. **b** and **c**. H460 cells expressing Bcl2 shRNA or control shRNA were treated with 100 μM H_2_O_2_ (**b**) and 200 μM NNK (**c**) for 60 min. Cells were then washed three times and incubated in fresh culture medium for indicated times. mtDNA damage or mtDNA mutation was analyzed as described in “Methods”. Quantification data are mean ± SD from three independent experiments

To test the physiological role of endogenous Bcl2 in regulating mtAPE1 activity, mtDNA repair and mtDNA mutation frequency, the relatively high levels of endogenous Bcl2 in H460 cells were depleted by RNA interference (RNAi) using Bcl2 shRNA as described in Materials and Methods. Transfection of Bcl2 shRNA significantly reduced the expression level of endogenous Bcl2 by more than 99 % in H460 cells (Fig. 6a). Control shRNA had no effect on Bcl2 expression. Intriguingly, specific knockdown of endogenous Bcl2 not only upregulated mtAPE1 endonuclease activity (i.e. increased amount of cleaved 14-mer product) but also accelerated mtDNA repair in association with deceased frequency of mtDNA mutations (Fig. 6). These findings provide strong evidence that physiologically expressed Bcl2 in cells is able to suppress mtDNA repair and promote mtDNA mutation through a mechanism involving the inhibition of mtAPE1 endonuclease activity.

## Discussion

Mitochondria are key intracellular organelles that serve as the powerhouse of eukaryotic cells. They are thus involved in critical processes deciding cell fate that are crucial for cell growth, survival and tumor development. Mitochondrial DNA (mtDNA) is remarkably vulnerable to oxidative or other genotoxic damage and displays a significantly higher mutation rate (10- to 200-fold) compared to the nuclear genome [54]. Numerous somatic mutations in both the coding and control regions of mtDNA have been extensively examined in a broad range of primary human cancers, underscoring the fact that accumulation of mtDNA mutations may be a critical factor in eliciting persistent mitochondrial defects and consequently contributing to cancer initiation and progression [55]. Intriguingly, accumulation of mtDNA mutations may also contribute to tumor metastasis [56]. However, the mechanisms of generating these mtDNA mutations in the carcinogenic process remain largely unknown, although it is known that mtDNA is subjected to continuous oxidative attack by free radicals [9].

NNK is one of the major carcinogens in tobacco. NNK has been associated with various cancers in tobacco users, especially lung cancer [57]. NNK can induce oxidative DNA damage, including the generation of AP sites in nDNA [58]. Our data show that, in addition to nDNA, NNK can also induce mtDNA damage and enhance mutation frequency in mtDNA (Figs. 1b and 2c). Intriguingly, mtDNA is more sensitive than nDNA to NNK-induced damage (Fig. 1b), suggesting that NNK-induced mtDNA damage and mutations may play a role in mtDNA-related diseases, including the development of various types of cancer.

Since the mtAPE1-mediated BER pathway is the main DNA repair route present in mitochondria [59], inhibition of mtAPE1-mediated mtDNA repair may lead to increased frequency of mtDNA mutations in mitochondria. Our findings reveal that expression of Bcl2 resulted in decreased mtAPE1 activity in mitochondria leading to suppression of mtDNA repair and accumulation of mtDNA mutations following exposure of cells to H_2_O_2_ or NNK (Fig. 2). Conversely, depletion of endogenous Bcl2 by RNAi enhances mtAPE1 endonuclease activity and accelerates mtDNA repair, which contributes to reduction of mtDNA mutations (Fig. 6). These findings identify a novel function of Bcl2. Bcl2 inhibition of mtDNA repair and enhancement of mtDNA mutations may promote tumorigenesis following exposure to carcinogens (i.e. NNK) or reactive oxygen species. Nuclear respiration factor 1 (NRF1) is the main factor regulating mitochondrial biogenesis and plays a crucial role in regulating the expression of a broad range of mitochondrial genes [60]. It has recently been reported that APE1 functions as a coactivator of NRF1 and regulates mitochondrial function through an NRF1-dependent pathway. Specific knockdown of APE1 impairs NRF1 DNA-binding activity [60]. Thus, Bcl2 inhibition of APE1 may also reduce NRF1 activity, which may partially contribute to decreased mtDNA level following H_2_0_2_ or NNK treatment. Further studies are required to demonstrate this possibility.

Mitochondrial localization is thought to be required for Bcl2 suppression of apoptosis and more than 90 % of Bcl2 is localized in mitochondria [36, 61]. Since mtBcl2 is co-localized and interacts with mtAPE1 in mitochondria (Figs. 3 and 4), this may explain how mitochondrial Bcl2 (mtBcl2) has inhibitory effects on mtAPE1 activity and mtDNA repair.

Bcl2 family members share homology in regions designated BH domains BH1, BH2, BH3, and BH4 [62]. All four BH domains are necessary for the robust antiapoptotic function of Bcl2 [28, 63, 64]. Since removal of any of these BH domains eliminates the effects of Bcl2 on mtAPE1 binding, mtAPE1 activity, mtDNA repair and mtDNA mutation (Figs. 4 and 5), this suggests that the interaction between Bcl2 and mtAPE1 in mitochondria is essential for Bcl2’s inhibitory effects on mtAPE1 activity and mtDNA repair and consequently for promotion of mtDNA mutations (Figs. 4 and 5). Thus, the oncogenic activity of Bcl2 may also require its BH domains.

## Conclusions

Here we have identified a previously unrecognized role of Bcl2 in regulating mtDNA repair and mtDNA mutation in human lung cancer cells. Bcl2 suppression of mtDNA repair occurs through its interaction with mtAPE1 in mitochondria via BH domains and subsequent suppression of mtAPE1 activity. Inhibition of mtDNA repair by Bcl2 in association with enhanced frequency of mtDNA damage may contribute to promotion of carcinogenesis and/or progression of cancer.

## Additional file

Additional file 1: **Supplemental data.** Supplemental Figures (**Figures S1-S4.**) are included. (PDF 403 kb)

## Abbreviations

mtDNA: mitochondrial DNA
NNK: nitrosamine 4-(methylnitrosamino)-1-(3-pyridyl)-1-butanone
mtAPE1: mitochondrial apurinic/apyrimidinic endonuclease 1
nDNA: nuclear DNA
ROS: reactive oxygen species
BER: base excision repair
DSBs: DNA double strand breaks
AP site: apurinic/apyrimidinic site
NHEJ: nonhomologous end joining
QD-IF: quantum dot-based Immunofluorescence
