# Supplementary material for: Bcl2 inhibition of mitochondrial DNA repair
Source: BMC Cancer. 2015 Aug 13;15:586. doi: 10.1186/s12885-015-1594-1 (PMC4535531; doi:10.1186/s12885-015-1594-1)
Supplement: Additional file 1: — Supplemental data. Supplemental Figures (Figures S1-S4.) are included. (PDF 403 kb) [file 12885_2015_1594_MOESM1_ESM.pdf]

Supplemental Data

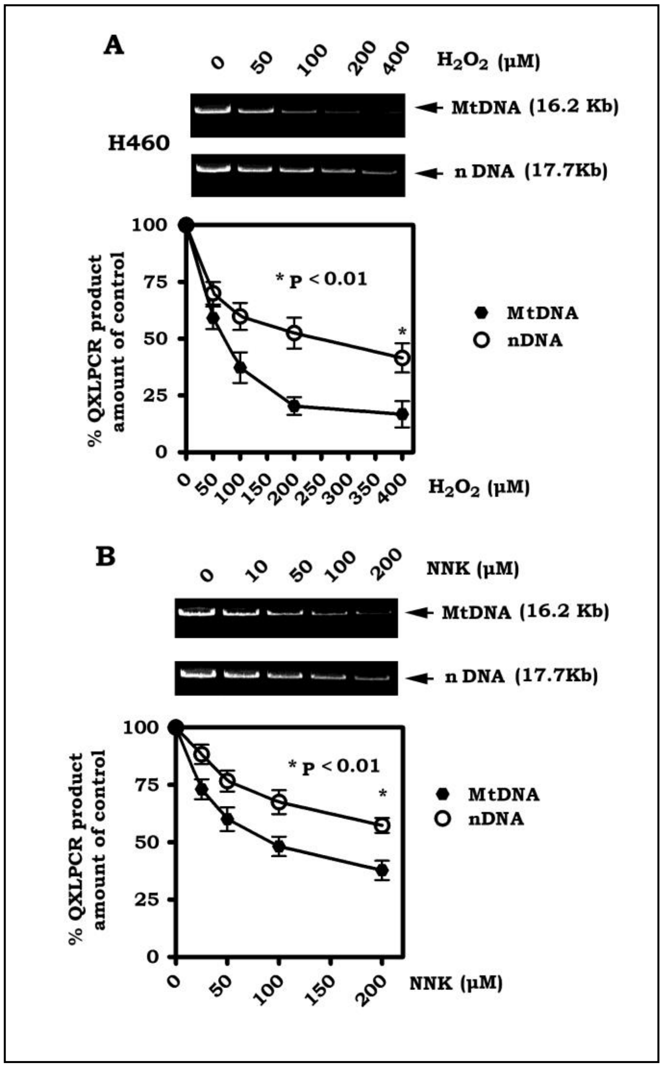

Figure S1. Mitochondrial DNA (mtDNA) is more vulnerable than nuclear DNA (nDNA) to damage induced by H_2O_2 or NNK in H460 cells. H460 cells were treated with increasing concentrations of H_2O_2 (**A**) or NNK (**B**) for 60 min. The mtDNA or nDNA damage was measured by QXLPCR and quantified by Pico Green dsDNA Quantitation kit. Quantification data are mean ± SD from three independent experiments.

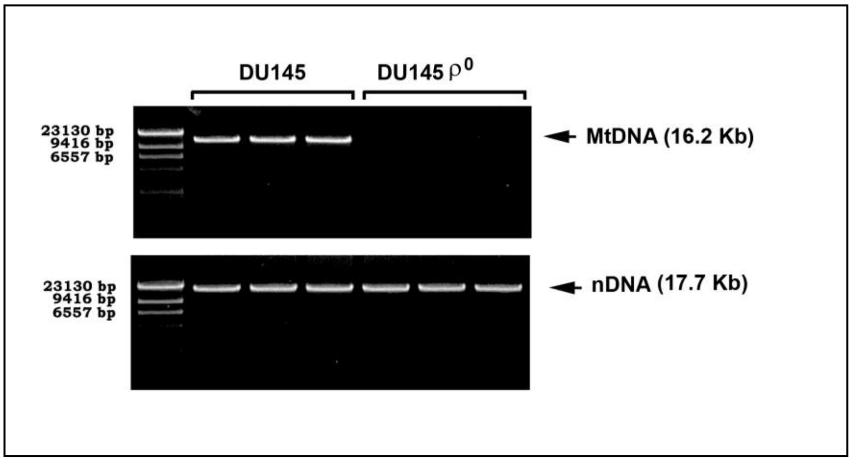

Figure S2. The mtDNA or nDNA was compared in DU145 cells and DU145 ρ^0 cells (mtDNA deficient cells) by QXLPCR using the primers for mtDNA or primers for the β-globin gene of nDNA, respectively, as described in "Methods".

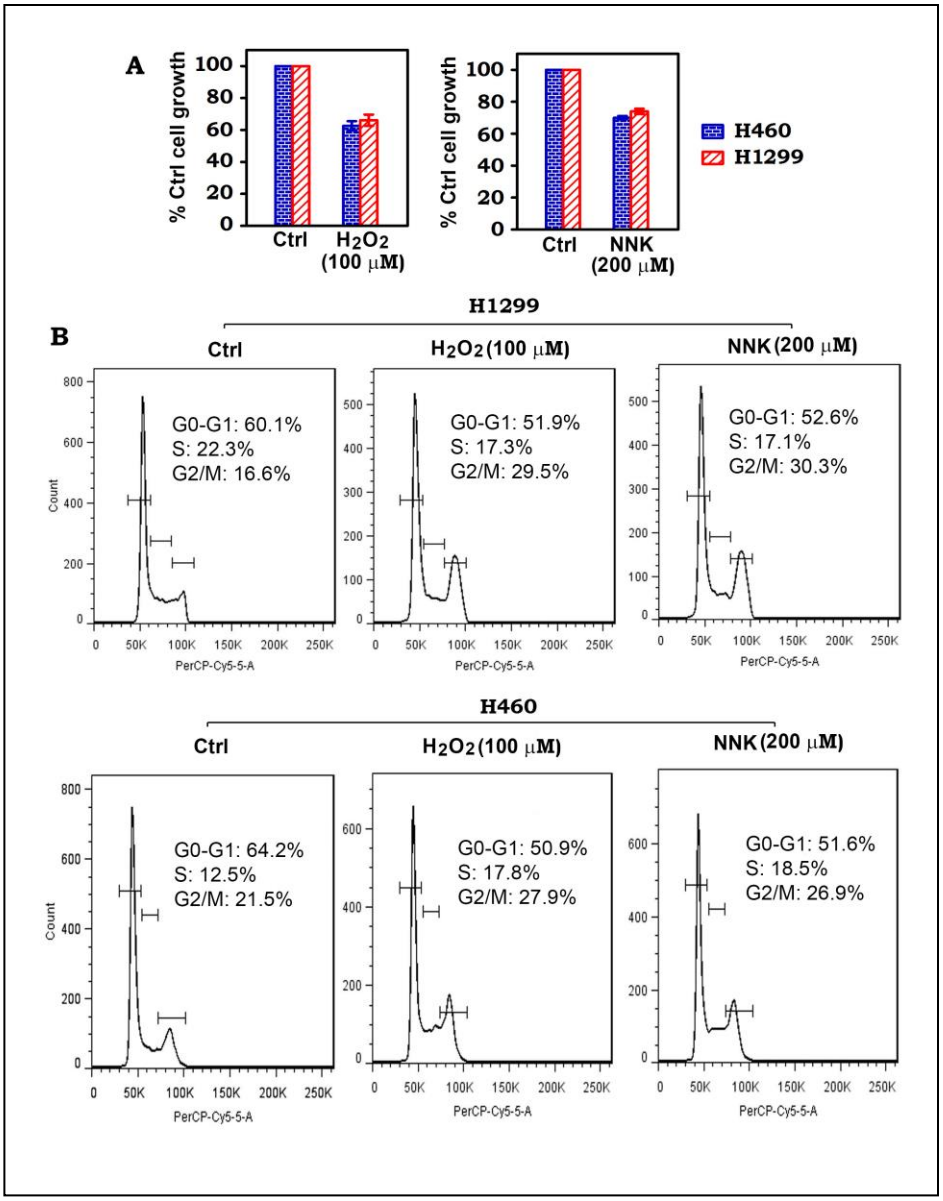

Figure S3. H1299 and H460 cells were treated with H_2O_2 (100 µM) or NNK (200 µM) in serum-free medium for 60 min, followed by washing with 1×PBS. Cells were cultured in normal medium for 24h. Cell proliferation was measured using MTT kit (**A**). Cell cycle was also analyzed as described in “Methods” (**B**).

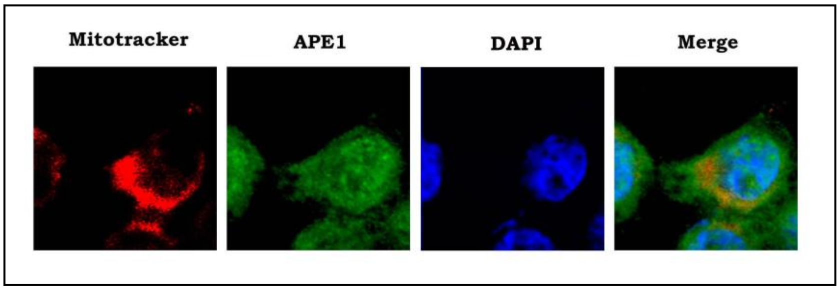

Figure S4. H460 cells were grown on LabTek 8-well chamber slides, then fixed with methanol and acetone (1:1) for 5 min at −20◦C. After blocking with 10% normal goat serum for 20 min at room temperature, chamber slides were incubated with rabbit APE1 primary antibody (1:500) overnight at 4◦C. After washing with PBS, the cells were incubated with Alexa 488 (green)-conjugated anti-rabbit and Mito Tracker (red, Invitrogen) for 30 min. After washing, cells were stained with DAPI for 60 min at room temperature and observed under a fluorescent confocal microscope (Zeiss, Sweden).
